# Machine learning assisted feature identification and prediction of hemodynamic endpoints using computed tomography in patients with CTEPH

**DOI:** 10.1007/s10554-023-03026-2

**Published:** 2023-12-24

**Authors:** Joshua Gawlitza, Sophie Endres, Peter Fries, Markus Graf, Heinrike Wilkens, Jonas Stroeder, Arno Buecker, Alexander Massmann, Sebastian Ziegelmayer

**Affiliations:** 1 Clinic/Institute of Diagnostic and Interventional Radiology, Klinikum rechts der Isar, Technical University of Munich, Ismaninger Straße 22, 81675 Munich, Germany; 2 Clinic for Diagnostic and Interventional Radiology, Saarland University Medical Center, Kirrberger Strasse 100 (Building 41), 66424 Homburg, Germany; 3 Cardiology, Angiology, Pulmonary and Intensive Care, Saarland University Medical Center, Kirrberger Strasse 100, 66424 Homburg, Germany; 4 Department of Radiology and Nuclear Medicine, University Hospital Schleswig-Holstein, Campus Lübeck, Lübeck, Germany; 5 Department of Radiology and Nuclear Medicine, Robert-Bosch-Krankenhaus, Auerbachstr. 110, 70376 Stuttgart, Germany

**Keywords:** CTEPH, Computed tomography, Pulmonary hypertension, Artificial intelligence

## Abstract

Chronic thromboembolic pulmonary hypertension (CTEPH) is a rare but potentially curable cause of pulmonary hypertension (PH). Currently PH is diagnosed by right heart catheterisation. Computed tomography (CT) is used for ruling out other causes and operative planning. This study aims to evaluate importance of different quantitative/qualitative imaging features and develop a supervised machine learning (ML) model to predict hemodynamic risk groups. 127 Patients with diagnosed CTEPH who received preoperative right heart catheterization and thoracic CTA examinations (39 ECG-gated; 88 non-ECG gated) were included. 19 qualitative/quantitative imaging features and 3 hemodynamic parameters [mean pulmonary artery pressure, right atrial pressure (RAP), pulmonary artery oxygen saturation (PA SaO2)] were gathered. Diameter-based CT features were measured in axial and adjusted multiplane reconstructions (MPR). Univariate analysis was performed for qualitative and quantitative features. A random forest algorithm was trained on imaging features to predict hemodynamic risk groups. Feature importance was calculated for all models. Qualitative and quantitative parameters showed no significant differences between ECG and non-ECG gated CTs. Depending on reconstruction plane, five quantitative features were significantly different, but mean absolute difference between parameters (MPR vs. axial) was 0.3 mm with no difference in correlation with hemodynamic parameters. Univariate analysis showed moderate to strong correlation for multiple imaging features with hemodynamic parameters. The model achieved an AUC score of 0.82 for the mPAP based risk stratification and 0.74 for the PA SaO2 risk stratification. Contrast agent retention in hepatic vein, mosaic attenuation pattern and the ratio right atrium/left ventricle were the most important features among other parameters. Quantitative and qualitative imaging features of reconstructions correlate with hemodynamic parameters in preoperative CTEPH patients—regardless of MPR adaption. Machine learning based analysis of preoperative imaging features can be used for non-invasive risk stratification. Qualitative features seem to be more important than previously anticipated.

## Introduction

Chronic thromboembolic pulmonary artery hypertension (CTEPH) is a subtype of pre-capillary pulmonary hypertension (PH) characterized by multiple chronic occlusive thrombi and emboli in the pulmonary arteries [1]. While its incidence ranges from 0.1 to 10% after acute pulmonary embolism (PE), recent evidence suggests even higher associations with acute PE, making CTEPH a frequently underdiagnosed condition [2, 3]. Right heart catheterization and ventilation/perfusion single photon computed tomography remain the cornerstone of CTEPH diagnosis. However, the value of computed tomography (CT), especially ECG-gated dual-source CT, is becoming increasingly recognized [4]. Notably, CT parameters have been identified that correlate strongly with mean pulmonary artery pressure (mPAP) [5]. Historical studies, dating back to 1984, established correlations between CT parameters and mPAP [6]. Despite this, inconsistencies persist in literature regarding the significance and methodology of various quantitative CT parameters [7, 8]. Moreover, current research often overlooks qualitative features, potentially missing vital imaging insights [9, 10]. While machine learning's potential in CTEPH diagnosis is largely unexplored, recent reviews highlight its prospective utility in PH imaging [11]. CTEPH patients in particular are in need of special diagnostics and therapy at certified centres, therefore large scale evaluation is difficult and the current scientific knowledge insufficient [1].

Our study seeks to bridge these gaps by comprehensively assessing the correlation of known quantitative and qualitative CT parameters with hemodynamic outcomes, considering their acquisition methods. Drawing from prior literature, we aim to provide a holistic overview and leverage machine learning to gauge the clinical relevance of various CT imaging features.

## Methods

### Study design

Data collection, processing and analysis were approved by the ethics committee of the university of Saarland University. The study was designed as a retrospective cohort study. Imaging and clinical parameters were collected by the department of diagnostic and interventional radiology. In total 127 Patients with previously diagnosed CTEPH were included in the study. CTEPH was defined as an increase in mean pulmonary arterial pressure (≥ 25 mm Hg at rest) due to persistent obstruction of the pulmonary circulation following pulmonary thromboembolism or DVT, which persists despite adequate anticoagulation, according to the definition of the European Respiratory Society [12]. Preoperative right heart catheterization and computed tomography angiography examinations of the thorax (with and without ECG-synchronization) were performed for every patient. Patients from the local university medical center were included over a time span of five years (2015–2020). All patients were evaluated as operable and underwent pulmonary endarterectomy following imaging and right heart catheterization. Inoperable patients, patients without right heart catheterization or marked imaging artifacts were excluded from this study.

### Imaging data acquisition

Patients were scanned on a third generation dual-source scanner Siemens Somatom Force (Siemens Healthineers, Erlangen, Germany). Computed tomography examinations of the thorax included ECG-gated studies performed to rule out coronary artery disease (39 patients) in a preoperative setting before pulmonary endarterectomy. ECG-synchronization included prospective triggering and retrospective gating depending on the heart frequency and rhythm. CT acquisition incorporated automated radiation exposure control with adjustments of tube voltage and current depending on the patient’s mass and body outline (CAREDose 4D, CAREkV, Siemens Healthineers, Erlangen, Germany) with basic image parameters being: reference tube voltage: 100 kVp, reference tube voltage: 288 mAs, collimation: 192 × 0.6. Image reconstruction included 0.6 and 1 mm axial slices at end-diastole using a soft tissue convolution kernel (Bv40) and advanced model iterative reconstruction (ADMIRE, Siemens Healthineers) at strength level 3.

In 88 patients CT examinations consisted in a dual-energy angiography study of the pulmonary arteries without ECG-synchronization using the following parameters: reference tube voltage: 90 kVp (tube A)/150Sn kVp (tube B), collimation 192 × 0.6). Accordingly, automated radiation exposure control was applied with reference tube currents set to 100 effective mAs (tube A) and 75 effective mAs (tube B). Image reconstruction included 1 mm axial slices using a soft tissue convolution kernel (Qr40) with advanced model iterative reconstruction (ADMIRE, Siemens Healthineers) at strength level 3.

All CTA studies were acquired with a single intravenous contrast agent bolus (Imeron 400, Bracco Imaging S.p.A., Milan, Italy) followed by a saline bolus administered with a double head power injector (Accutron CT-D, Medtron AG, Saarbrücken, Germany). Contrast agent volumes ranged from 60 to 90 mL with flow rates ranging from 3.5 and 5 mL/s depending on the purpose of the CT scan as well as patient´s weight and the size of the venous access device.

### Imaging variables and hemodynamic parameters

**Fig. 1:**
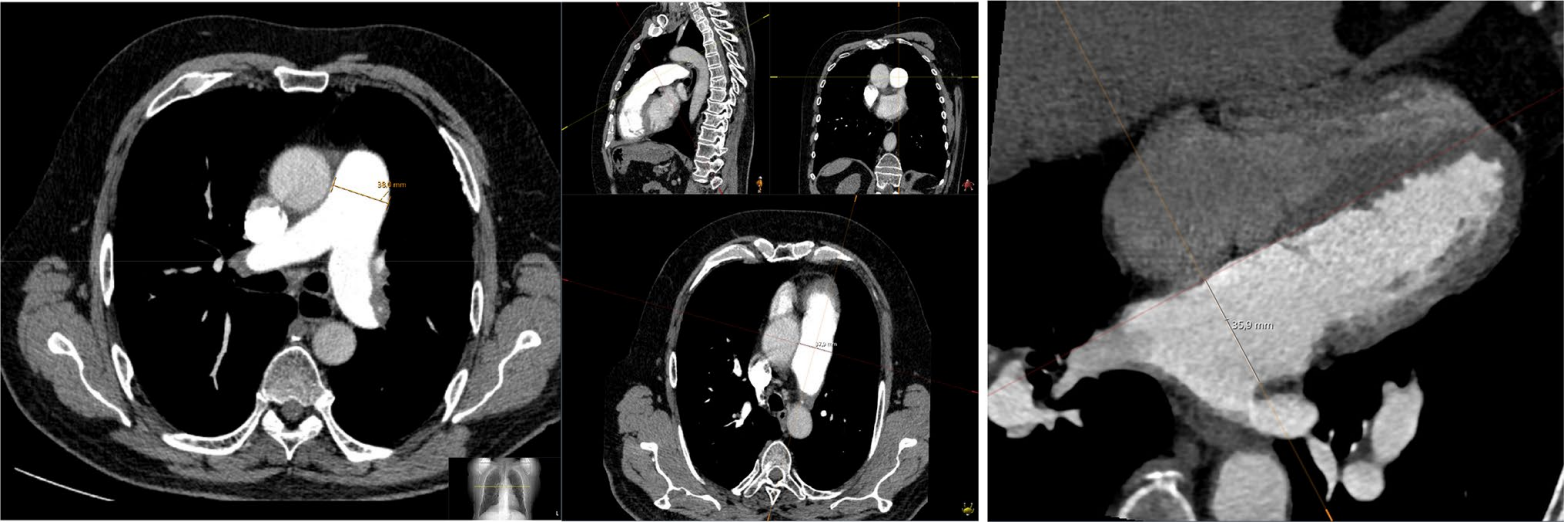
Measurements of computed tomography parameters in axial plane and multiplanar reconstruction. Shown is the different measurement technique in simple axial (left) and multiplanar reconstruction (right) for the same parameter—pulmonary trunc diameter. Difference in pulmonary trunc diameter was 0.1 mm in this patient. Further, the exemplary measurement of the left atrium in MPR is show

**Fig. 2:**
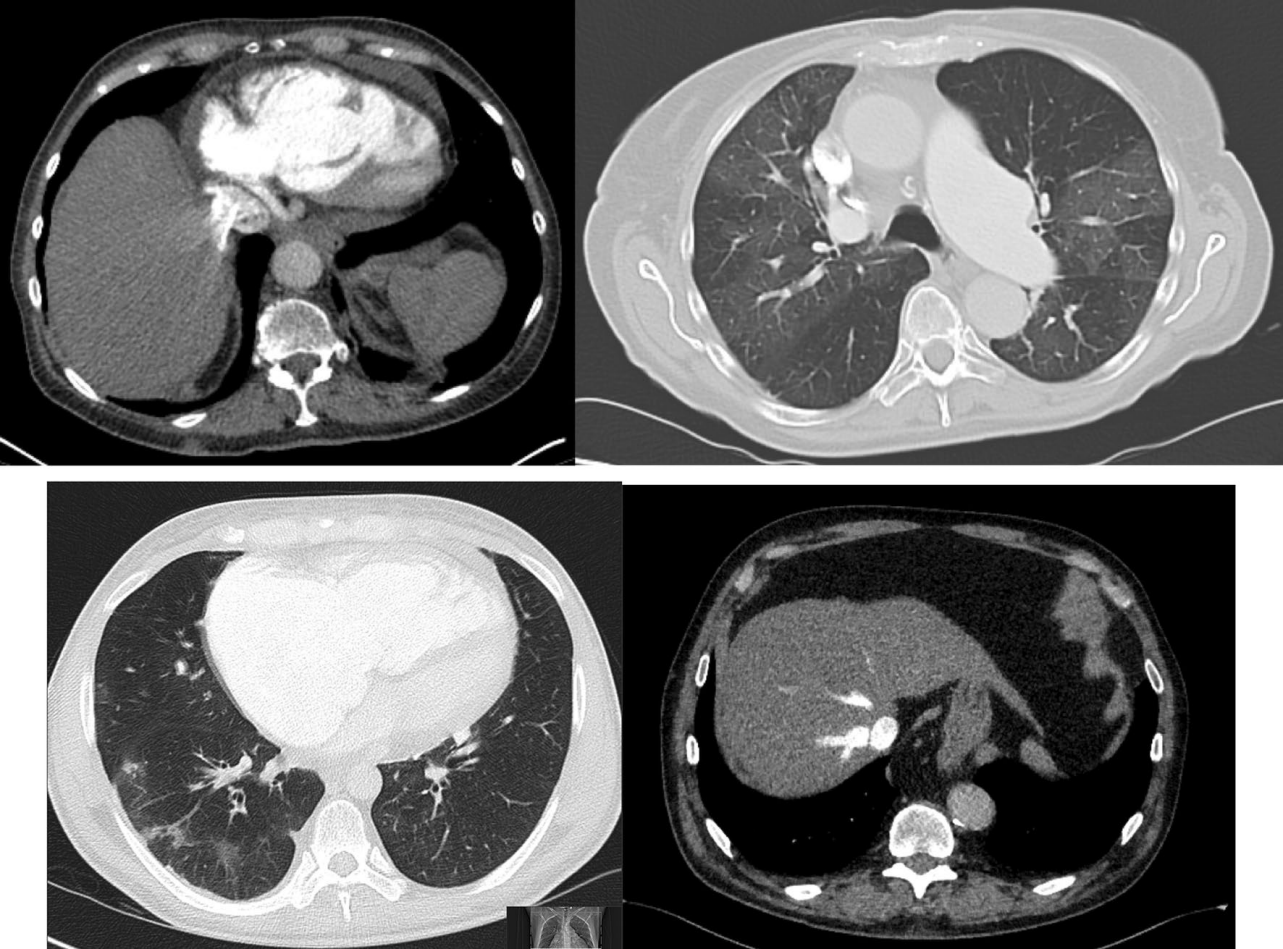
Exemplary images of qualitative findings. Shown are exemplary images of the different qualitative findings (from upper left to lower right): pericardial effusion, mosaic perfusion, groundglass opacities, contrast media retention in hepatic veins

We surveyed technical research on CTEPH imaging to identify and summarize known parameters. Consequently, the following quantitative parameters were measured in the CT: diameter of pulmonary trunk, left/right pulmonary artery (PA) and ascending aorta (AAo). Further, short axes of both left and right atrium (LA/RA) and ventricle (LV/RV) were measured. All diameters and short axes measurements were acquired once on the axial reconstructed image and once in the adjusted multiplanar reconstruction as exemplarily shown in Fig. 1. Three ratios were calculated from the named parameters: ascending aorta diameter/pulmonary trunk diameter, left ventricular diameter/right ventricular diameter and right atrial diameter/left ventricular diameter. According to previous literature, the ventricle septum angle, septum thickness and left/right ventricle area were measured as well [11, 13]. Both axes and area measurements were performed as cavity measurements, having the borders of the measurements defined by the endocard. Beyond the quantitative parameters, qualitative parameters were included as well, including contrast media reflux in the inferior caval vein, contrast media retention in the hepatic veins, pericardial effusion, mosaic perfusion as well as intrapulmonary ground-glass opacities. Exemplary images can be found in Fig. 2. In total 14 quantitative and 5 qualitative imaging features were measured, calculated or acquired in the reconstructed planes described above and depending on the feature in either axial or multiplanar reconstruction (MPR). Measurements were performed once, but supervised by a second radiologist and corrected, if necessary.

For the qualitative features, the presence or absence of hepatic and cava reflux of contrast, mosaic attenuation, pericardial effusion and ground-glass opacity were assessed by two board-certified radiologists in a blinded fashion. In case of disagreement, a consensus decision was reached in retrospect. Hepatic and inferior cava reflux was assessed using the semi-quantitative methods described by Groves et al. [14]. Groves scale 3 (reflux into the inferior cava vein but not the hepatic veins) and Groves scale 5 (reflux into the IVC and opacifying the midpart of the hepatic veins) were used to properly separate between the two discrete findings. Although often appearing together in CTEPH, mosaic perfusion and ground-glass opacities were included regardless of their size and distribution (e.g. regional versus peri-arteriolar).Right heart catheterization was performed at the local university hospital, usually via cubital vein using a Swan-Ganz balloon tipped catheter. Our study focused on three parameters, acquired by right heart catheterization: the mean pulmonary artery pressure (mPAP), which was measured for all 127 patients. Further, the right atrial pressure (RAP), which was available for 101 patients and oxygen saturation of the pulmonary artery (sPO2), which was available for 55 patients. Clinical parameters (sex and age) were collected for all patients.

### ML modelling and statistical analysis

Statistical analysis and machine learning modelling was conducted using *Python* (Version 3.9.5), *Scikit-learn* (Version 1.1) and JMP (SAS, USA). Univariate analysis was performed by computing the coefficient of determination (R-squared) for quantitative features and calculating the student's t-test for qualitative features. Firstly, all features were compared between the different acquisition protocols (ECG, non-ECG gated). Both measurement methods (reconstructed multiplanar planes vs. standardized axial planes) were tested for correlation and outcome prediction. A p value of ≤ 0.05 was considered to be statistically significant.

For the ML-modelling the quantitative features were normalized to the (0,1) interval. Based on Cannon et al. a cut-off of 38 mm Hg was chosen to define high risk and low risk patients [15]. Additionally, a cut-off of 60% was chosen for the PA SaO2. A random forest algorithm was trained on the respective groups for both classification tasks. For testing, 3-folded random sampling was used to evaluate the model. Lastly, fast correlation-based filtering, a technique to prove model stability by reducing the feature set down to the most relevant and robust parameters, was used to compute the individual feature importance [11, 16].

## Results

Comparison of ECG- and non-ECG-guided CT showed no significant differences in quantitative and qualitative characteristics and for further analyses both groups were merged.

### Axial versus MPR measurement

Based on the measurement methods, significant differences were found for 5 features: left and right PA diameter, AAo diameter, RV diameter and LA diameter [p = 0.0129 to < 0.0001 (Table 1)]. Differences in absolute values of the above-mentioned features were below 0.3 mm. When looking at the endpoint prediction (mPAP, RAP, sPO2) no significant differences were found between axial and MPR measurements.

**Table 1:** Absolute differences of measurement in axial and MPR measurements

	Axial mean measurements (mm)	MPR measurements (mm)	Mean difference (mm)	p value
Mean pulmonary trunc	3.44	3.43	0.007	0.499
Mean left pulmonary artery	2.62	2.64	0.019	0.0129*
Mean right pulmonary artery	2.77	3.06	0.29	< 0.0001*
Mean asc. Aorta	3.37	3.54	0.17	< 0.0001*
Short axis left atrium	3.83	3.93	0.1	0.0004*
Short axis right atrium	5.80	5.83	0.03	0.47
Short axis left ventricle	4.07	4.07	0.002	0.89
Short axis right ventricle	5.11	5.31	0.2	< 0.0001*
Septum thickness	0.89	0.89	0.002	0.8

*=significant

**=highly significant

### Predictive power of univariate imaging variables

Univariate analysis revealed multiple parameters significantly correlating with the hemodynamic outcomes. The best predictive power for each hemodynamic parameter is as follows:

mPAP—left PA diameter r = 0.49,

RAP—short axis RA r = 0.5,

PA SaO2—ratio RA/LV r = − 0.51.

**Fig. 3:**
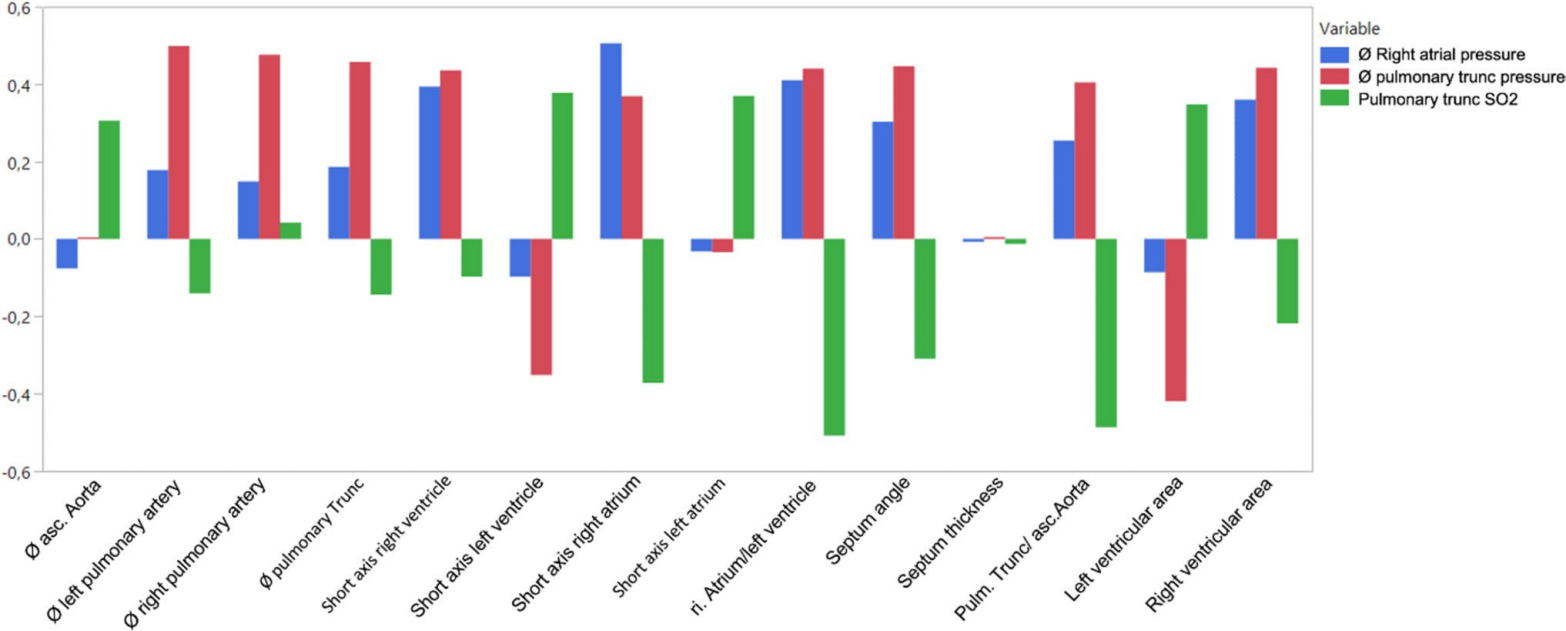
Coefficients of determination for all quantitative features with respect to the hemodynamic parameters. Measurement ratios show moderate to strong correlation with all three hemodynamic parameters. Shown are the correlations strengths of the quantitative computed tomography parameters and the hemodynamic parameters from right heart catheterization. Strongest correlations were found for: pulmonary trunc pressure—left pulmonary artery diameter (r^2^ = 0.49), right atrial pressure—short axis right atrium (r^2^ = 0.5) and pulmonary trunc SaO2—ratio right atrial diameter/left ventricle diameter (r^2^ = − 0.51)

All coefficients of determination for both measurement methods are depicted in Table 2. Regarding the hemodynamic parameters evaluated, the ratio-based features tend to be superior representations compared to the measurement-based features, exemplified by the coefficient of determination of the left PA diameter compared to the RA/LV-ratio for RAP, mPAP and PA SaO2 of 0.18, 0.49, − 0.14 and 0.41, 0.44, − 0.51, respectively. The corresponding coefficients of determination for all quantitative features and hemodynamic parameters are shown in Fig. 3.

**Table 2:** Correlations between computed tomography measurements and angiography pressures

	Mean asc. Aorta	Mean left PA	Mean right PA	Mean pulmonary Trunc	Short axis right ventricle	Short axis left ventricle	Short axis right atrium	Short axis left atrium	ri. Atrium/left ventricle	Septum angle	Septum thickness	Pulm. Trunc/ asc.Aorta	Left ventricular area	Right ventricular area
Ø Right atrial pressure	− 0.08	0.18	0.15	0.19	0.39**	− 0.1	0.5**	− 0.03	0.41**	0.3**	− 0.007	0.25*	− 0.09	0.36**
Ø pulmonary trunc pressure	0.005	0.49**	0.48**	0.46**	0.44**	− 0.35**	0.37**	− 0.03	0.44**	0.45**	0.01	0.41**	− 0.42**	0.44**
Pulmonary trunc SO_2_	0.31*	− 0.14	0.04	− 0.14	− 0.1	0.38**	− 0,37**	0.37**	− 0.51**	− 0.31*	− 0.01	− 0.49**	0.35**	-0.21

Shown are the correlation coefficients (r) between the three measured pressure values and the computed tomography features

*PA* pulmonary artery, *SO2* oxygen saturation

*Significant correlation (p value ≤ 0.05)

**Highly significant difference (p value ≤ 0.001)

For the qualitative features contrast media retention in the hepatic veins and inferior vena cava as well as pericardial effusion showed significant differences for mPAP, PA SaO2 and RAP. Interestingly in regard of contrast media retention in the hepatic veins highly significant differences were found in all three cases (p value = 0.001 and < 0.0001). In comparison contrast media retention in the vena cava inferior displayed highly significant differences just for RAP (p value = 0.0005) and pericardial effusion altogether only showed mildly significant differences for the three hemodynamic parameters (p-value between 0.0023 and 0.0447). Mosaic attenuation indicated highly significant differences for both RAP and mPAP (p value = 0.0009 and < 0.001).

**Fig. 4:**
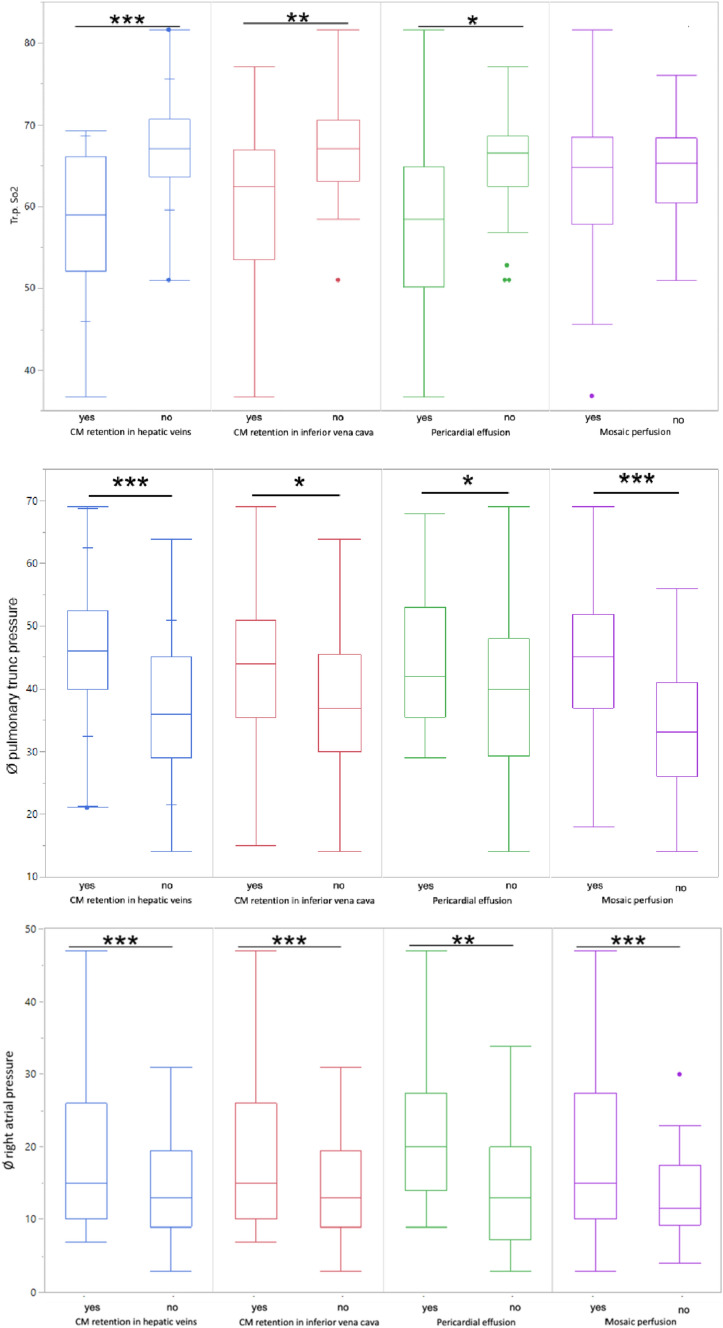
Boxplots in regard to qualitative features. Shown are the differences in qualitative features regarding right atrial pressure, pulmonary trunc pressure and pulmonary trunc partial oxygen pressure. Highly significant differences are marked with *** and significant differences with *

Overall the most significant differences (p ≤ 0.0001) were found for the appearance of contrast media retention in hepatic veins for mPAP and PA SaO2. Boxplots displaying the differences for the qualitative features for each hemodynamic parameter are shown in Fig. 4.

### Non-invasive risk stratification using ML-models

Using random sampling for algorithm assessment on the test dataset the random forest reached an AUC of 0.82 (sensitivity of 0.97; specificity of 0.52, positive predictive value of 0.92) for the binary prediction of the mPAP and 0.74 (sensitivity of 0.90; specificity of 0.50, negative predictive value of 0.81) for the prediction of the PA SaO2. Fast correlation-based filtering yielded four highly important features (Table 3). For the binary mPAP prediction the RA/LV ratio, mosaic attenuation pattern, left PA diameter and contrast retention in hepatic veins were the most important features in descending order. Similarly, contrast retention in hepatic veins, MP/AAo ratio, pericardial effusion and the RA/LV ratio were the most important features in descending order for the binary prediction of the PA SaO2.

**Table 3:** Differences in qualitative findings in regard to angiographic parameters

	SpO_2_ (%)			Mean pulmonary trunc pressure			Mean right atrial pressure		
	Yes	No	p value	Yes	No	p value	Yes	No	p value
Contrast media retention in hepatic veins	58	67	< 0.0001**	46	37	< 0.0001**	11	7.5	0.001**
Contast media retention in inferior vena cava	61	67	0.003*	42	37	0.0349*	10.7	7.2	0.0005**
Pericardial effusion	58.1	65.2	0.0447*	44	39	0.0403*	12	4.3	0.0023*
Ground glass opacities	60.5	64	0.3102	43	39.7	0.21	10.9	8.6	0.21
Mosaic perfusion	62.7	64.2	0.4727	44.9	33.4	< 0.001**	10.5	7.1	0.0009**

Shown are the differences in angiographic parameters between the different qualitative features

*=significant

**=highly significant

## Discussion

In our study, we demonstrate the correlation of different quantitative and qualitative imaging features with hemodynamic parameters in preoperative patients with CTEPH and show that although measurement methods (axial vs MPR) may differ significantly with respect to the imaging features, both the absolute value and the correlation with the hemodynamic parameters are not significantly affected. We found that several quantitative features show moderate to strong correlation with the hemodynamic parameters and that qualitative features were able to significantly differentiate hemodynamic endpoints. Of particular note is the contrast retention in the hepatic veins and inferior vena cava, which, beside pericardial effusion, show significant differences for all hemodynamic endpoints and may be a morphological correlate for right heart dysfunction [17]. Lastly, an ML model was used for a non-invasive risk stratification of the patients, with relevant features being both quantitative and qualitative. Our study harnesses machine learning to surpass traditional univariate analysis by simultaneously interpreting complex feature interactions for enhanced diagnostic accuracy, as substantiated by our robust, cross-validated random forest model.

The correlation between hemodynamic parameters and CT features has been heavily investigated as described above. Recent studies, for example by Swift et al. [18], have focused on etiological independent pulmonary hypertension and included non-ECG-gated CT examinations. Roller et al. analysed a heterogeneous cohort of 45 CTEPH patients with ECG-gated CT scans [19]. In our study, we included 127 CTEPH patients all of whom underwent PE and CT, to facilitate a homogeneous and standardised analysis. No significant differences in quantitative and qualitative characteristics were found between ECG-gated (n = 39) and non-ECG-gated (n = 88) CT scans, which is in line with current literature [20, 21]. Furthermore, we included qualitative imaging features and extended the analysis to RAP and PA SaO2 as two additional hemodynamic endpoints. While the conventional diagnosis of CTEPH requires RHC, V′/Q′ scan and a pulmonary angiography, a current statement paper of the European respiratory society discusses the use of dual-source CT (DSCT) as an alternative method [22]. In particular, for a proximal CTEPH manifestation, DSCT seems to be a sufficient alternative [23]. Our results confirm the utility of CT in CTEPH and demonstrate the potential for a non-invasive prediction of the mPAP, RAP and PA SaO2. The evaluation of the axial and the reconstructed measured features showed that although significant differences exist for individual features, there were no relevant differences in accuracy for the univariate analysis and the ML model. Consequently, the quantitative and qualitative features can be measured from readily available axial image slices. Similar results were described for cardiac measurements.

Although we were able to prove not only the importance of qualitative features, but gave an overview regarding available quantitative CT features including predictive power, several limitations must be addressed. Although measurements have been reevaluated by a second, experienced reader, measuring the quantitative features multiple times and averaging them, might have provided an even higher data quality. Due to the limited size and retrospective mono-institutional nature of our study, the ML-model may not generalize to unseen data. Although 127 CTEPH patients is respectable in regard, that only three hospital centres in Germany are specialized for this disease, further studies are required to test the capability of ML-models for this task. We have constrained the algorithm's input data to observable or measurable features and used a decision tree-based architecture. The rapid development of neural networks, especially convolutional neural networks, is achieving outstanding results in the medical computer vision field. Accordingly, the utility of CNNs in the hemodynamic endpoint prediction using the image data should be investigated. Although a significant correlation between imaging parameters and hemodynamic outcomes has been demonstrated in a preoperative group of patients with CTEPH, the benefits for patient management in operable cases remain unclear and need to be evaluated.

## Conclusion

In our study we were able to show through a generalized overview of correlations between quantitative and qualitative imaging features with hemodynamic parameters in CTEPH patients the importance of individual features. Especially the significance of qualitative features, such as contrast retention in hepatic veins, have been underestimated in the past. ML models trained on the quantitative CT features allow a basic risk stratification of preoperative patients, making non-invasive preoperative evaluation conceivable in future clinical applications.

## Data Availability

The data underlying this article will be shared on reasonable request to the corresponding author.
